# Working Together: Contributions of Corpus Analyses and Experimental Psycholinguistics to Understanding Conversation

**DOI:** 10.3389/fpsyg.2018.00525

**Published:** 2018-04-12

**Authors:** Antje S. Meyer, Phillip M. Alday, Caitlin Decuyper, Birgit Knudsen

**Affiliations:** Max Planck Institute for Psycholinguistics, Radboud University, Nijmegen, Netherlands

**Keywords:** conversation, turn-taking, language production, speech planning, polar questions

## Abstract

As conversation is the most important way of using language, linguists and psychologists should combine forces to investigate how interlocutors deal with the cognitive demands arising during conversation. Linguistic analyses of corpora of conversation are needed to understand the structure of conversations, and experimental work is indispensable for understanding the underlying cognitive processes. We argue that joint consideration of corpus and experimental data is most informative when the utterances elicited in a lab experiment match those extracted from a corpus in relevant ways. This requirement to compare like with like seems obvious but is not trivial to achieve. To illustrate this approach, we report two experiments where responses to polar (yes/no) questions were elicited in the lab and the response latencies were compared to gaps between polar questions and answers in a corpus of conversational speech. We found, as expected, that responses were given faster when they were easy to plan and planning could be initiated earlier than when they were harder to plan and planning was initiated later. Overall, in all but one condition, the latencies were longer than one would expect based on the analyses of corpus data. We discuss the implication of this partial match between the data sets and more generally how corpus and experimental data can best be combined in studies of conversation.

## Introduction

As far as we know, conversation exists in all cultures and is the most common way of using language (Levinson, 2016). Because of the obvious social importance of conversation, language scientists should study its properties and the cognitive processes making it possible. Much of our current knowledge about conversation is based on analyses of corpora of recorded everyday conversations. These studies have led to important insights into the linguistic properties and structure of conversation. However, we argue in this paper that the cognitive processes occurring in the interlocutors’ minds can only be fully understood when they are also studied through experimental research. The main aim of the present paper is to illustrate how corpus analyses and experimental psycholinguistics can complement each other in research on conversation.

To showcase the proposed approach we focus on a key feature of conversation, the rapid transitions between turns. To account for this observation, Levinson and Torreira (2015) proposed that interlocutors begin to plan their utterances as early as possible, often while still listening to the interlocutor. As we explain below, this early-planning hypothesis can be tested far better in the lab than through corpus analyses. To illustrate the experimental approach, we review three relevant studies. Their results largely supported the early-planning hypothesis, but also showed that the participants’ speech onset latencies were much longer than expected on the basis of the corpus analyses. This finding is problematic because the aim of the experiments was to understand how short gap durations arise in conversation. We propose that the discrepancy may, at least in part, be due to the fact that corpus analyses and laboratory studies concerned different types of utterances. To assess this proposal, we conducted two experiments where participants produced the same kind of utterances, namely answers to polar questions, as in one of the relevant corpora of conversational speech. Polar questions are questions such as “Would you like a drink?” to which an affirmative or negative answer (e.g., “yes” or “no”) is expected (e.g., Holmberg, 2013). The main research question was whether the gap durations would be as short as those observed in conversational corpora. To anticipate the results, this was only the case in one of several experimental conditions. The theoretical and methodological implications of these findings are considered in the section “General Discussion.”

In conversations, interlocutors take turns in speaking and listening. Turns are on average about two seconds long, but there is much variation in their lengths, from about a third of a second to several hours (Sacks et al., 1974; Stivers et al., 2009; Levinson and Torreira, 2015; Levinson, 2016). The length of the turns is not pre-planned, nor is the sequence of speakers in multi-party conversations. In spite of this variability, turns are tightly coordinated in time. Usually only one person talks and the gaps between turns are short. As Levinson (2016) puts it, “the system [of turn taking] is highly efficient: less than 5% of the speech stream involves two or more simultaneous speakers (the modal overlap is less than 100 ms long), the modal gap between turns is only around 200 ms, and it works with equal efficiency without visual contact” (p. 6). The tight coordination between turns has been seen as suggesting a general human tendency to synchronize or align with others (e.g., Hari et al., 2015).

How do interlocutors manage to coordinate their turns so well? Most of the time, they cannot do so by simply reacting to the end of their partner’ s turn. This is because turns often overlap and the most common gaps, with modal durations around 200 ms (Stivers et al., 2009; Heldner and Edlund, 2010), are too short to plan an utterance. Picture and action naming studies have shown that planning a single content word takes at least 600 ms (e.g., Indefrey and Levelt, 2004), and initiating a description of an event often takes well over a second (e.g., Griffin and Bock, 1998; Konopka and Meyer, 2014). Although much of this planning time is taken up by the visual and conceptual processing of the picture and it is unknown how long speakers need to formulate their own thoughts in the absence of pictorial input, these data nevertheless show that speakers cannot plan an utterance within 300 ms. In fact, even launching a fully specified utterance plan typically takes around 400 ms (Ferreira, 1991; Wesseling and van Son, 2005; Piai et al., 2011). In order to achieve smooth transitions between their turns, interlocutors must therefore begin to plan their utterances while listening to their partner and launch them shortly before the end of the partner’s turn (De Ruiter et al., 2006; Magyari and De Ruiter, 2012; Pickering and Garrod, 2013; Clark and Lindsey, 2015; Garrod and Pickering, 2015; Riest et al., 2015).

Levinson and Torreira (2015) have proposed a theoretical framework capturing the coordination of listening and speaking in conversation. They assume that upcoming speakers can often identify the current speaker’s speech act (whether it is, for instance, a statement, request, or question) and the gist of the utterance well before the end of the turn. For example, a customer in a restaurant seeing a waiter approaching with a bottle of wine saying “Do you ...?” can quickly guess the waiter’s intention and respond accordingly. Levinson and Torreira (2015) propose that upcoming speakers begin to plan their utterance as soon as they have sufficient information to do so. This is usually when they have identified the speech act and gist of the partner’s utterance. Meanwhile, they continue to listen, use the incoming information to predict the end of the current turn, and when it is close, prepare the articulators and initiate the utterance.

Levinson and Torreira’s (2015) early-planning hypothesis explains the short gaps and occasional overlap between turns. However, from the perspective of cognitive psychology it is surprising that interlocutors would opt for such early planning. This is because there is ample evidence that listening and speech planning both require cognitive capacity (Strayer and Johnston, 2001; Ferreira and Pashler, 2002; Kemper et al., 2005; Cook and Meyer, 2008; Becic et al., 2010; Roelofs and Piai, 2011; Cleland et al., 2012). Carrying out these processes in parallel should be cognitively demanding, and one might expect interlocutors in a conversation to try to minimize rather than maximize their mental workload.

If the early-planning hypothesis is correct, gap durations should be shorter when utterance planning can be initiated early compared to when it can only be initiated late during the preceding turn. This prediction cannot be evaluated very well through corpus analyses because it is often difficult to establish on the basis of transcripts of conversations when the information the upcoming speaker needed for their response was conveyed. Moreover, gaps in conversation are likely to depend on many other factors as well, for instance on the type of turn planned, its complexity, and the interlocutors’ speech rates (e.g., Roberts et al., 2015). Therefore, subtle effects of the onset of planning may be hard to detect through corpus analyses. The early-planning hypothesis can more readily be assessed in experiments where the timing of the response-relevant information can be controlled and the effect on gap durations can be established. To illustrate this approach, we review three relevant studies.

The first study was an EEG study by Bögels et al. (2015b, see also Bögels et al., 2018). Participants answered quiz questions where the cue to the answer was either presented relatively early, as in “Which character, also called 007, appears in the famous movies?” or at the very end of the question as in “Which character from the famous movies is also called 007?” Participants were 310 ms faster to answer in the early than in the late cue condition. Moreover, in the EEG signal a late positivity starting around 500 ms after cue onset was observed in both conditions. This positivity was reduced in a control condition where participants had to remember, but not answer the questions. Based on the scalp distribution of the positivity, the authors concluded that the late positivity was related to planning of the phonological form of the utterance. These results show that participants started planning their answers as soon as they had enough information to do so.

In the second study, Barthel et al. (2016) obtained similar evidence using a list-completion paradigm. Here, a participant and a confederate, both native speakers of German, saw sets of objects on their screens. The confederate named her items first, and the participant then named any additional items they saw on their screen. Importantly, the confederate’s description either ended in a noun or a verb form [e.g., “Ich habe eine Puppe und einen Schuh (besorgt),” “I have a doll and a shoe (obtained)”]. The participants’ speech onset latencies were shorter in the verb-final than in the noun-final condition. This shows that they began to plan their utterances as soon as they had heard the final noun and knew which objects the confederate could see and name, and which object names they had to add to the list. Consistent with the early-planning hypothesis and with the data obtained by Bögels and colleagues, participants already began to plan their utterances in the verb-final condition while listening to the final word of the confederate’s turn (for related evidence see also Barthel et al., 2017).

In a third study, Sjerps and Meyer (2015) used a dual-task paradigm with eye tracking to assess when participants began to attend to response-relevant information. Participants saw displays featuring two rows of four objects each. In the critical condition, they first heard a recorded sentence referring to one of the two rows of objects (“Put the hat under the chair and put the apple above the tree”) and then had to describe the objects in the other row in the same way. Their eye movements were recorded to track how long they would look at each row of objects. In one condition, the participants in addition carried out a continuous tapping task measuring their mental workload. The results showed that at the beginning of the trial, the participants primarily looked at the objects mentioned in the recorded sentence, but approximately a second before the end of the recording, they began to look primarily at the objects they had to name themselves. Around the same time, their tapping performance deteriorated, which indicates an increase in mental load (see also Boiteau et al., 2014). Thus, as in the studies by Bögels and colleagues and by Barthel and colleagues, participants began to plan their utterances while still listening to the other person.

However, contrary to the prediction derived from Levinson and Torreira’s framework, they did not look at the relevant objects as early as possible. This would have been as soon as they had heard the first noun in the recording and therefore knew which row was being described and which row they would have to describe themselves. In sum, the three studies support the view that speech planning begins before the end of the preceding turn, but they do not provide consistent support for the view that speakers begin to plan their utterances as early as possible.

A noteworthy finding of all of these studies is that the participants’ speech onset latencies were considerably longer than the typical gaps in conversation. In the dual-task condition of the study by Sjerps and Meyer (2015) described above the average latency was 390 ms, and it was 329 ms in a control condition where participants did not have to tap while preparing their utterances. The latencies in the study by Bögels and colleagues were 640 and 950 ms for the early and late cue condition, respectively; and the corresponding latencies in the study by Barthel and colleagues were 749 and 842 ms, respectively. The long response latencies in these studies may seem potentially problematic because the latencies were regarded as equivalent to gaps in conversation, and a central research goal was to understand how the gaps in conversations can be as short as they are.

There are many reasons why the latencies could have been longer than gaps in conversation. One possibility is that in natural conversations, speakers can often begin to plan their utterances even earlier than in the experiments. Another likely reason is that utterance planning is often easier and less time consuming in conversation than in the experiments. The participants in the study by Bögels et al. (2015b) had to search their long-term memory for answers to quiz questions concerning, for instance, names of actors or European capitals and produce the appropriate proper names. In the study by Barthel et al. (2016), they had to scan complex displays and establish which objects had already been mentioned and which still needed to be named. Finally, in the study by Sjerps and Meyer (2015), the participants had to plan lengthy utterances, and they may have postponed the onset of planning to minimize the interference between the nouns they heard and the nouns they had to select themselves. Situations similar to those realized in the experiments arise in natural conversations as well, but often responding to a turn may be considerably easier and should therefore be associated with much shorter response latencies. For instance, many turns in conversation consist of acknowledgments and back-channeling, such as “uhm,” “uhuh,” which, due to their broad semantic meaning, frequency, and simple phonological form, should be easy to plan (e.g., Roberts et al., 2015). In short, in conversation, there is likely to be much variation in when planning can begin and how time-consuming it is, but since many utterances can be planned early and are easy to generate, the average gap duration is short. By contrast, the experiments elicited relatively hard utterances (answers to quiz questions and fairly long sentences), which therefore lead to longer latencies compared to the gaps in conversations.

This informal comparison between the experimental results and corpus data illustrates a simple point: When corpus and experimental work are meant to address the same empirical question (e.g., when speakers begin to plan utterances), they should concern comparable utterances. Although this requirement seems obvious, it is not easy to realize. This is because available corpora are often not large enough or annotated well enough to extract sufficient numbers of relevant turns and because it may not be obvious which properties of the turns are relevant when studying a specific question.

Nevertheless, researchers drawing on both experimental and corpus work should strive, as much as possible, to compare like with like. The present study is meant to illustrate and discuss how this might be done. A corpus that is much cited in work on gaps in conversation is the corpus from Stivers et al. (2009), which focusses on a single type of adjacency pairs, polar (yes/no) questions, and their answers. The authors investigated the gaps between polar questions and answers in 10 structurally different languages. They found that across all languages, polar questions were typically answered with gaps around 200 ms, though there was considerable variation around these means both within and across languages. For Dutch, which is the language studied in the present research, the authors found an average gap duration of 109 ± 69 ms (95% confidence interval, based on 224 observations, see Supplementary Materials to Stivers et al., 2009).

This corpus, though small, targeted a specific type of adjacency pairs and constitutes a good basis for comparison with results of a laboratory study of the same type of adjacency pairs. In the present study, we asked Dutch participants to answer polar questions about objects they could see on their screen and examined whether the average response latency would fall within the confidence interval of the average gap duration in the Dutch corpus of polar questions. If this is the case, the experiment succeeded in creating a scenario similar to those that allow speakers to respond fast in everyday conversations. This would indicate that the experimental paradigm can be used to study how interlocutors process and plan utterances in this particular setting. Furthermore, the results can be used to derive new hypotheses about the ways speakers may process and plan utterances in different settings, for instance, when talking about more complex arrays or producing different types of utterances.

**FIGURE 1:**
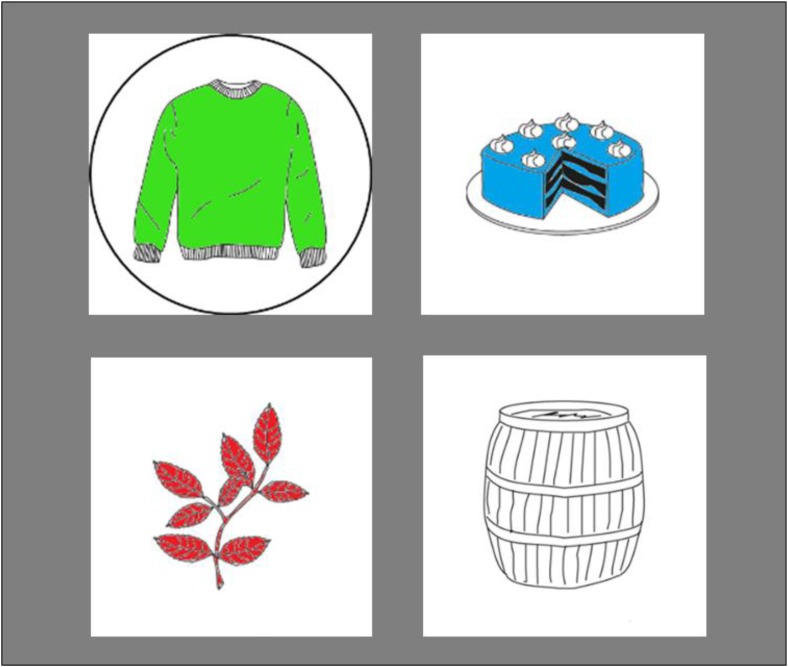
Example display used in Experiments 1 and 2. The circle appeared only in Experiment 2 on Participant Q’s screen.

In Experiment 1, participants saw displays featuring four colored line drawings and heard questions referring to the color of one of the objects (**Figure 1**). For instance, they might see a cake, a branch, a sweater, and a barrel and hear a question such as “Heb je een groene trui?” (“Do you have a green sweater?”). The same set of objects was used on all trials of a block (see the section “Materials and Methods”). The participants knew that the target object would always be included in the display but could appear in the color mentioned in the question or in a different color. They answered as quickly as possible, saying “ja” (“yes”) or “nee” (“no”). The response latencies were recorded.

There were two display conditions: In the monochrome condition, all objects had the same color. Therefore, the participants could begin to plan their response as soon as they had understood the color adjective. The adjective began on average 731 ms before the end of the question. Since only four adjectives with different onset consonants were used, the participants could begin to plan their response as soon as they had identified the initial consonant of the adjective, roughly 500 ms before the end of the question. If they indeed chose to plan their answer that early, they should be able to respond within about 100 ms after the end of the question. By contrast, if they postponed utterance planning, for instance until they had heard the entire question, much longer latencies should be observed.

In the second condition, the multi-color condition, the four objects had different colors. The participants therefore had to wait for the noun and they had to check whether the object appeared in the color specified in the question. For instance, to answer the question “Do you have a green sweater?” they had to find the green object and determine whether or not it was a sweater. An alternative, probably less efficient strategy would be to search for the sweater and determine whether or not it was green.

This condition was primarily included to create a task that was not all too trivial for the participants. Yet, the comparison of the latencies in the monochrome and multi-color condition was of some interest as well. The latencies should be longer in the multi-color than in the monochrome condition. Obtaining such a result would demonstrate that the experimental paradigm is suitable to study not only how speakers create short gaps between their turns, but also how variation in gap durations arises. Although much of the literature has highlighted the short durations of the typical gaps between turns there is considerable variation around these modes. Thus, while strikingly short gap durations are a hallmark of conversation, variation of gap durations is an important feature of conversation as well and needs to be explained.

In Experiment 1, participants were tested individually and answered pre-recorded questions. In Experiment 2, they worked in pairs and took turns asking questions about the partner’s display and answering the questions. This experiment additionally included a variation of the length of the required responses: They were either simply “yes” or “no” as in Experiment 1, or they included a comment on the location of the object, as in “yes, in the top left position,” or on its color, as in “no, I have a red one.” One aim of Experiment 2 was to replicate the main findings of Experiment 1 in a laboratory context that came closer to everyday conversations by including a conversational partner. A second aim, further discussed below, was to explore the effect of the length of the response on the speech onset latencies.

## Experiment 1

### Method

#### Participants

Twenty-one native speakers of Dutch (mean age 22 years, *SD* = 3 years; 10 males) took part in the experiment. They were recruited from the participant pool of the Max Planck Institute for Psycholinguistics in Nijmegen. All participants were students of Radboud University and reported having normal color vision and no speech or language disorder. They were paid for participating in the study. Ethical approval for the study had been given by the Ethics Board of the Social Sciences Faculty of Radboud University. Data from one female participant were excluded due to equipment failure.

#### Materials

The visual stimuli were four sets of four colored line drawings of everyday objects (see Appendix A in the Supplementary Materials and **Figure 1**). The drawings were taken from the picture gallery of the Max Planck Institute for Psycholinguistics. Dutch nouns have neutral or non-neutral gender and the grammatical gender of the noun is marked in the affix of most color adjectives in noun phrases including the indefinite article [e.g., “een groen huis,” “a green house” (neuter) versus “een groene bal,” “a green ball” (non-neuter)]. All picture names used in the present study had non-neuter gender so that the adjective form did not provide a cue to the upcoming noun. The names of the pictures within a set shared the initial consonant (/t/, /b/, /k/, or /p/) so that participants in the multi-color condition had to listen to most of the noun before planning the response. The pictures appeared in four colors, blue, green, red, and white. They were scaled to a size of 10 by 10 cm, corresponding to 9.50° of visual angle for the participant. They appeared centered in the four quadrants of the screen.

On each trial the participant heard a recorded question, produced earlier by a trained native speaker of Dutch enquiring about the color of one of the objects in the set. All questions had the same structure, namely “Heb je een [color adjective][noun]?,” as in “Heb je een groene trui?” (“Have you a green sweater?”). As there were 16 objects and 4 colors, there were 64 different questions. The average duration of the question was 1137 ms (*SD* = 73 ms). The color adjective began on average after 406 ms (*SD* = 36 ms), and the noun after 779 ms (*SD* = 51 ms).

#### Design

There were four test blocks of 64 trials each. In each block one of the four sets of objects was used. The order of the blocks was counterbalanced across participants. The spatial arrangement of the four objects varied randomly from trial to trial, with each object appearing equally often in each position.

In each block there were 32 trials featuring the monochrome condition and 32 trials featuring the multi-color condition. The two trial types appeared in random order. There were four different random lists, each of which was presented to five participants. Each color was used on eight of the monochrome trials. On multi-color trials the four objects differed in color. Thirty-two pseudo-random combinations of colors and objects were used such that across the multi-color trials within a block each object appeared eight times in each color.

The questions associated with each display were chosen to elicit positive and negative responses on half the trials in each condition. Each color and object was mentioned equally often. All participants were presented with the same combinations of displays and questions.

#### Apparatus

Audacity^®^ software (version 2.0.6, Audacity Team, 2014) was used to create the auditory stimuli. The experiment was run on a desktop computer using Presentation^®^ software (version 16.5, NeuroBehavioral Systems Inc., 2013). Voice recordings were made within Presentation with a Sennheiser ME64 microphone. Praat software (Boersma and Weenink, 2017) was used to determine the speech onset latencies off-line, measured from the offset of the question.

#### Procedure

Participants were instructed before the first block. The relevant pictures were introduced at the beginning of each block on an instruction screen showing the drawings and their names. The participants were asked to listen to the questions and respond as quickly as possible, saying “ja” or “nee.”

The trial structure was the same in both conditions. A trial began with the presentation of the four pictures, which stayed in view until the end of the trial. One second after picture onset the question was presented. As soon as the participant responded the pictures were replaced by a blank screen. For the purpose of controlling the experiment, verbal response onsets were recorded using a voice key. The question was always presented in full, regardless of when the participant responded. The next trial began 1.50 s after the onset of the response or, when a response was given before the offset of the question or when no response was registered, 1.50 s after the end of the question.

#### Analyses

The participants’ responses were categorized as correct or incorrect. Incorrect responses were rare in all conditions (<3% of the trials) and were excluded from the analyses of response latencies. As no timeout for responses was set *a priori*, excessive response times were eliminated by removing responses with latencies over 2.5 standard deviations from the mean by condition. This affected between 0 and 3% of the correct trials per condition. Response times were not log-transformed because the outlier-removal procedure addresses the problematic long-tails the log-transform is typically used to address. Additionally, negative response latencies occurred (when participants responded before the end of the question), which would introduce additional complexities into the log-transform (see also Heldner and Edlund, 2010).

The naming latencies were analyzed in R (R Core Team, 2017) using the lme4 package (Bates et al., 2015b) for mixed-effects models, the car package (Fox and Weisberg, 2011) for ANOVA-style overviews, and the effects (Fox, 2003) and ggplot2 (Wickham, 2009) packages for effects displays. For model coefficients, we interpret |*t*| > 2 to correspond to significance at the 5% level (via the convergence of the *t*-distribution to the normal for large samples, cf. Baayen et al., 2008). Effects displays show 83% confidence intervals. Non-overlap of these intervals corresponds to the 5% significance level of the difference.

There were two categorical variables: display type (monochrome or multi-color) and response type [positive (“ja”) or negative (“neen”)]. They were sum coded, so that model coefficients represent main and not simple effects. In other words, the intercept term represents the weighted grand mean and the coefficients (βs) represent the offset from the intercept (or relevant main effect in the case of interactions) and thus half the offset between conditions (because the grand mean is equidistant from each condition). There were no continuous predictors. Random effects were chosen to be maximal without overparameterization in order to best balance Type-I error and power (Barr et al., 2013; Bates et al., 2015a; Matuschek et al., 2017). As such, there were by-participant and by-item random intercepts as well as by-participant and by-item random slopes for all main effects, but not for interactions. There were 64 items (16 objects each appearing in four colors). All models were fit with maximum-likelihood estimation (i.e., REML = FALSE). The final model is reported in Appendix B in the Supplementary Materials.

### Results and Discussion

**FIGURE 2:**
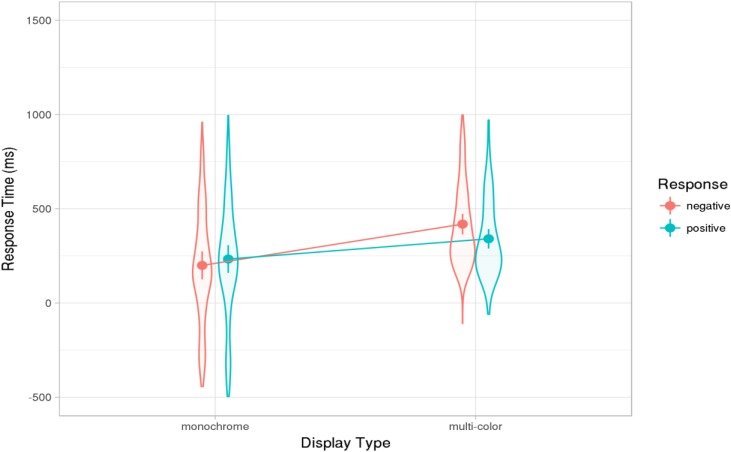
Results of Experiment 1: Effect estimates (grand-mean response latencies computed from the mixed models, ms) for positive and negative responses in monochrome and multi-color displays. Error bars show 83% confidence intervals; non-overlap indicates significance of the difference at the 5% level (see text). Violin-plot overlay indicates the distribution of the data included in the analysis.

The average response latencies per condition are shown in **Figure 2**. The main goal of the study was to examine whether the response latencies in the monochrome condition, where planning could begin about 500 ms before the end of the question, would correspond to the typical gap durations observed for polar questions in Dutch conversations. This turned out to be the case. The average latency was 215 ms, with the 95% confidence interval ranging from 111 to 321 ms and overlapping with the confidence interval of 40–178 ms reported for the corpus of Dutch polar questions by Stivers et al. (2009). The average latency of just over 200 ms indicates that the participants began to plan their utterance well before the end of the question, in line with the early-planning hypothesis discussed above.

As expected, the average response latency was significantly faster in the monochrome than in the multi-color condition (β = -82, *t* = -5.85). In the monochrome condition, the response-relevant information was presented earlier than in the multi-color condition (with the presentation of the adjective rather than the noun) and the visual and conceptual processes preceding the responses were less complex. Thus, the latency difference between the conditions is not surprising.

No predictions had been made about any differences in the latencies for positive and negative responses. In the corpus examined by Stivers et al. (2009), answers coded as confirming the question (positive answers, 81% of 224 responses) were associated with shorter gaps than answers coded as disconfirmations (negative answers), though this difference was not statistically significant for Dutch (for further discussion of the properties of confirming and disconfirming answers see also Bögels et al., 2015a; Kendrick and Torreira, 2015). Consistent with this observation, positive responses in our experiment were overall slightly faster than negative ones, yielding a significant main effect of response type (β = -11, *t* = -2.38). In addition, there was an interaction of response type and display type (β = -28, *t* = 5.26). As **Figure 2** shows, in the multi-color condition, negative responses were much slower than positive ones, whereas there was a small difference in the opposite direction in the monochrome condition. How this interaction arose is not clear. It may be related to decision or monitoring processes. In the very easy monochrome condition, participants made positive and negative decisions fast and with equal confidence; after all, they only had to decide whether the color shared by all objects in the display matched the adjective in the question. In the multi-color condition, they had to carry out a conjunction search task, establishing, for instance, whether or not the display included an object that was both green and a sweater. Both positive and negative answers could, in principle, be given as soon as either the sweater or the green object had been identified. One might speculate that participants sequentially inspected the objects and stopped the search process and provided a positive response as soon as they encountered the target in the correct color, but tended to inspect all objects before providing a negative answer (though the search could have been stopped as soon as they had encountered either the target object or the object in the target color). Such double-checking of the decision may be related to some reluctance to provide a negative response.

## Experiment 2

In Experiment 2, participants were tested in pairs and took turns asking and answering the same questions as in Experiment 1. Now the response latencies were indeed gaps between turns. As before, the person answering the question (Participant A hereafter) saw either a monochrome or a multi-color display. As for Experiment 1, the main research question was how the response latencies would compare to the gap durations reported in the Dutch corpus of polar questions by Stivers et al. (2009). In addition, we expected to replicate the effect of display type, with participants responding faster in the monochrome than in the multi-color condition and the effect of response type, with positive responses given faster than negative ones. A new manipulation introduced in Experiment 2 concerned the length of participant A’s utterances. In the short-response condition, participants said “ja” or “nee,” as in Experiment 1. In the new long-response condition, they provided additional information about the objects on their screen: In positive responses, they specified the location of the target object as in “ja, in de positie links boven” (“yes, in the top left position”). In negative responses, they specified the color of the target object, as in “nee, ik heb een rode sweater” (“no, I have a red sweater”). Intuition suggests that such qualified answers, in particular for negative responses, might be more consistent with the conventions of everyday conversation than simple “yes” or “no” answers (for an extensive discussion of the variables affecting the timing of preferred and less preferred responses, such as accepting and rejecting offers and requests in conversation, see Kendrick and Torreira, 2015). Thus, the long-response condition might approximate everyday conversations better than the short-response condition.

The long responses consisted of two parts, the initial “ja” or “nee” and the following phrase or sentence. If the participants planned these two parts of their utterances separately and began to speak as soon as they had planned the first part, the response latencies should be similar to those in the short response condition or even shorter, as the long responses were pragmatically more appropriate. In addition, there should again be a substantial difference between the responses given in the monochrome and multi-color condition.

Alternatively, the speakers might opt for planning the full utterance, at least at the conceptual level, before speech onset. In that case, the response latencies should be longer in the long-response than in the short-response condition. Moreover, there should be little difference between the monochrome and multi-color condition because in both conditions, participants had to identify the target and its position or color before responding. Thus, the experiment allowed us to study how participants planned the two-part responses.

### Method

#### Participants

Sixty-eight native speakers of Dutch (34 pairs) took part in this experiment. They were recruited from the participant pool of the Max Planck Institute for Psycholinguistics and were paid for taking part in the study. Eight pairs had to be excluded due to equipment failure. One pair was excluded because one of the participants reported after the experiment that she was colorblind, one pair because one of the participants’ speech was unintelligible on most trials, and four pairs because they nodded or shook their heads on a large number of trials instead of saying “ja” or “nee.” The data reported below are based on the results obtained from 20 pairs (mean age = 21 years, *SD* = 2; nine males).

#### Materials and Design

As noted, participants were tested in pairs and took turns in posing questions, assuming the role of Participant Q, and answering the questions, assuming the role of Participant A. Since the individual trials were longer than in Experiment 1, the number of trials per block was reduced from 64 to 40, yielding 20 responses per block from each participant.

For Participant A, 160 of the displays of Experiment 1 were used. For Participant Q, 160 new multi-color displays were created using the same line drawings as used for Participant A. In each of Participant Q’s displays, a circle was drawn around the object in the top left position, indicating that the participant should ask a question about this target object. All objects appeared as targets equally often (10 times per block). Targets appeared equally often in each of the four quadrants on A’s screen.

As in Experiment 1, one set of four objects with alliterating names was used per block, and the order of the four blocks was counterbalanced across participants using a Latin Square design. For participant A, half of the trials featured monochrome and half multi-colored displays. The two trial types appeared in random order. Participant Q always saw a multi-colored display. The experimental blocks were preceded by a 10-trial practice block, featuring five monochrome and five multi-colored displays on Participant A’s screen. All 16 line drawings appeared on the practice trials.

In addition to the display type, the type of response to be provided by Participant A was varied between participants. Ten pairs of participants were asked to provide short responses, “ja” or “nee.” The remaining 10 pairs were asked to give longer answers, specifying the positions of the targets in positive responses, and the actual colors of the target objects in negative responses. In positive responses, participants should say “ja, op de positie linksboven/rechtsboven/linksonder/rechtsonder” (“yes, in the top left/top right/bottom left/bottom right position”). In negative responses, they should say “nee, maar ik heb een” [color adjective, target name], as in “nee, maar ik heb een groene trui.” (“no, but I have a green sweater”).

#### Apparatus

The experiment was run on two HP Elite 8540p laptops using Presentation^®^ software (NeuroBehavioral Systems Inc., 2013). The laptops were connected via a PC-Link device that enabled the Presentation scripts on both laptops to run simultaneously and interactively. Recordings were made with a Roland R44 recorder with two Sennheiser ME64 microphones. The participants sat opposite to each other and could therefore not see each other’s screen.

#### Procedure

Participants were led to the lab, introduced to each other, and instructed together. They were given a booklet showing all line drawings and colors that would appear in the experiment. Each participant was asked to name aloud all pictures while the other person and the experimenter listened. The participants were told that they would play a game, and that Participant Q should try to guess the color of the target object on Participant A’s screen. Participant Q should ask a question about any of the colors used in the game except for the color of the target object on their own screen. This was because the objects never appeared in the same color on both screens. Participant A should truthfully answer the questions. The participants were given examples of the expected question and answer formats. After the instructions, they practiced the task on 10 trials, and then the experiment commenced.

A trial began with the simultaneous presentation of the displays on the participants’ screens. Then Participant Q asked a question and Participant A answered. One of the participants, randomly selected by the experimenter before the onset of the experiment, was asked to move the experiment forward by pressing the space bar when an answer had been given. Two-hundred milliseconds later the next trial began.

#### Analysis

The participants’ utterances were transcribed by a trained native speaker. Praat software (Boersma and Weenink, 2017) was used for acoustic analyses of the utterances. As in Experiment 1, the speech onset latencies (measured from the offset of the question) for correct responses were used as the dependent variable for the statistical analysis. Statistical methods were as in Experiment 1 except that response length (long or short) was included as a categorical predictor in addition to display type (monochrome or multi-color) and response type (positive or negative). Twice as many negative as positive responses were expected because Participant Q guessed the color of the target object on Participant A’s screen and had a 1:3 chance of guessing correctly. The actual rate of negative responses was 66%, in line with guessing at chance level.

The response latencies of Participant A were analyzed using the same method and packages as in Experiment 1. Response latencies deviating by >2.5 SD from the condition mean were excluded from the analyses (2% of the valid trials, between 0 and 3% per condition). Again, response times were not log-transformed. Random effects were chosen to be maximal without overparameterization. There were by-participant and by-item random intercepts as well as by-participant and by-item random slopes for all main effects except response length, but not for interactions. Response length was omitted because it showed near perfect correlation with other random slopes, which is indicative of overfitting, and removing it from the model had a trivial effect on the model fit.

Although the research questions concerned Participant A’s answers, rather than Participant Q’s questions, trials on which Participant Q named the target object incorrectly or hesitated within the utterance were excluded. This concerned 8% of the trials, with no condition losing more than 10% of the trials. The average duration of the remaining questions was 1366 ms (*SD* = 480 ms) with the adjective appearing on average 676 ms (*SD* = 429 ms) and the noun 1018 ms (*SD* = 468 ms) after the onset of the question.

### Results and Discussion

Participant A’s answers in the short-response condition almost always had the expected format (bare “ja” or “nee”) and were correct, with error rates varying between 1 and 3% across conditions. Answer formats in the long-response condition were more varied. Specifically, 53% of the positive answers had the expected structure as in “ja, in de positie links boven.” On 12% of the trials, the definite article “de” was omitted, and on a further 20% of the trials, the whole phrase “in de positie” was omitted, yielding, for instance, “ja, links boven.” On 8% of the trials participants produced longer answers, as in “ja, ik heb een groene trui in de positie links boven.” (“yes, I have a green sweater in the top left position”). On 58% of the negative responses, participants used the expected structure as in “nee, maar wel een groene trui.” On 7% of the trials, the contrasting conjunction “maar wel” was dropped or changed to “wel.” On 11% of the trials (mostly from one participant), elliptic utterances were used as in “nee, maar wel een groene.” (“no, but green one”). On 24% of the trials, participants added a verb phrase as in “nee, maar ik heb een groene trui” (“no, but I have a green sweater”). Participants were not corrected during the experiment in order to maintain the character of a fairly natural interaction. Since all of these utterances were longer than bare “yes” or “no” answers, they were included in the analyses. We only excluded answers that mentioned incorrect screen positions or colors. This was the case for 2% of all valid responses, varying between 0 and 3% across conditions.

**FIGURE 3:**
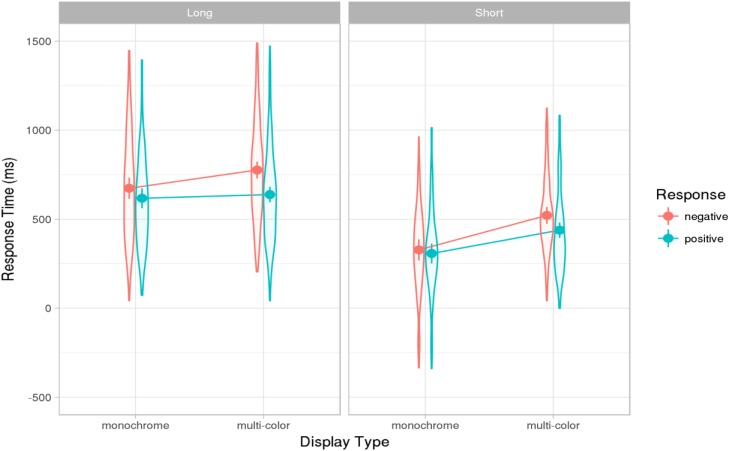
Results of Experiments 2: Effect estimates (grand-mean response latencies computed from the mixed model, ms) for long and short positive and negative responses in monochrome and multi-color displays. Error bars show 83% confidence intervals; non-overlap indicates significance of the difference at the 5% level (see text). Violin-plot overlay indicates the distribution of the data included in the analysis.

The mean latencies in each condition are shown in **Figure 3**. Note that the 95% confidence interval for the average latency in the fastest condition – short answers in the monochrome condition (240–402 ms) – did not overlap with the confidence interval of 40–178 ms for the gaps in the Dutch corpus by Stivers et al. (2009). This finding is further discussed below.

The final mixed effects model for the effects of response length and response type is shown in Appendix C in the Supplementary Materials. Overall short answers were given faster than long ones, yielding a significant main effect of response length (β = 139, *t* = 5.93). This indicates that the participants did not consistently initiate the long responses as soon as they knew that the first part of the utterance should be “ja” of “nee,” but rather carried out at least some of the planning for the second part of the utterance before responding. Furthermore, responses were given significantly faster in the monochrome than in the multi-color condition (β = -56, *t* = 7.97). There was an interaction between response length and display type, resulting in a decrease in the monochrome advantage for long responses (β = 25, *t* = 3.59). In the monochrome condition, it was easier for participants to decide whether the answer should be positive or negative, but conceptualizing and formulating the second part of the long answers were not facilitated in the monochrome condition. Therefore, participants planning long utterances benefited less from monochrome compared to multi-color displays than participants planning short utterances. These results indicate that participants did not plan the long utterances in the most incremental fashion, initiating the response as soon as they knew what the first part of the answer was, but instead began to plan the second part before responding. This conclusion is supported by the observation that participants rarely paused between the first and the second part of the answer: Pauses longer than 200 ms, which can be seen as planning pauses, occurred on only 18% of the valid trials of the long answer type.

As in Experiment 1, negative responses were given more slowly than positive ones (β = 37, *t* = -5.57; **Figure 3**). There was an interaction between response type and display type, with the monochrome display type benefiting less from the advantage for positive responses (β = -18, *t* = -4.58). No further interactions with response length were significant. This pattern is consistent with the findings of Experiment 1: Positive responses were given faster than negative ones, except in the easiest condition (short answers in the monochrome condition), where positive and negative responses were given with equal speed (see Appendix D in the Supplementary Materials for a joint analysis of the results of both experiments).

## General Discussion

In order to understand the cognitive processes occurring when language is used in conversation both careful analyses of corpora of natural conversations and experimental investigations are needed. We argued in the section “Introduction” that these research approaches best complement each other when, informally speaking, like is compared with like, i.e., when experimental research and corpus analyses target the same utterances or turns.

We illustrated this approach by comparing the gap durations in a small Dutch corpus of polar questions and answers to the latencies for responses to polar questions elicited in the lab. An important question was whether these latencies would approximate the gap durations in the corpus. Such equivalence would suggest that the experiment realized one of the scenarios that allow speakers in natural conversations to respond to such questions with short gaps. This in turn would mean that the experimental paradigm can be used in further research into speech planning in conversations. To put this differently, if the experimental paradigm is to be used to study how short gaps between questions and answers arise in natural conversation, it would be good if these short latencies could be elicited in the lab.

As reported above, in the monochrome condition of Experiment 1, the participants responded as fast as anticipated on the basis of the corpus data, but they failed to do so in the multi-color condition of that experiment and in all conditions of Experiment 2. This suggests that characteristics of natural conversations that allow speakers to respond swiftly were not realized in most of the experimental conditions, or that some properties of the experimental setting encouraged or forced speakers to respond more slowly than they do in natural conversations.

On the basis of the available evidence it is impossible to determine what these characteristics might be. In the present study, the participants’ utterances referred to pictorial displays changing from trial to trial, and the participants had to carry out a visual search to find the target. By contrast, in natural conversations interlocutors sometimes talk about their environment and need to find objects their interlocutor are referring to before responding (e.g., “What’s that bird on the birch tree? A parrot?”); but often they talk about events in the past or future and refer back to earlier parts of their conversations. The sensory and conceptual processes involved in deciding how to answer polar questions in everyday conversations must be highly variable and must often be quite different from the processes involved in generating the utterances in our study. The social situation in the lab was, of course, also different from most everyday interactions. In Experiment 1, the participants responded to recorded sentences, and in Experiment 2 they interacted with a stranger. Although similar situations occur in everyday life, other communicative situations may be more common, and there may be motivational and pragmatic factors encouraging speakers to respond earlier in everyday conversations than they did in the present study. Little is known about the interlocutors recorded in the corpus by Stivers et al. (2009) and about the contexts they interacted in. The authors merely report that they “collected videotaped interactions of maximally informal, spontaneous, naturally occurring conversations, each with 2–6 consenting participants” and that “the participants were often engaged in additional activities (e.g., eating, drinking, or stringing beads)” (p. 1059). Most likely they often knew each other and occasionally, but not in all of their interactions, referred to their shared environment. In our experiments, we created scenarios that resembled everyday communicative situations in certain ways, but that certainly deviated in many ways from most of the natural settings where the corpus data were recorded. How strongly the specific features of our scenarios affected the participants’ speech onset latencies is unknown, but it is possible that shorter (or longer) average latencies might have been obtained if we had elicited the utterances in a different way or if we had used a different social setting.

In spite of the imperfect match between the predictions and the experimental findings, the project illustrates how corpus-based research and experimental research can complement each other. The corpus analyses suggested a specific hypothesis about latencies for answers to polar questions. This hypothesis was only confirmed in one of the two experiments and, as noted, only for one condition. This partial success (or failure) can be seen as an incentive for further theoretical and experimental work and targeted corpus analyses into the conditions that enable interlocutors in conversation to respond to each other with the observed short gaps.

A similar point can be made concerning the response length effect observed in Experiment 2: Short answers were given faster than longer ones, demonstrating that the participants producing long responses planned more than the first word before beginning to speak. If this is also true for everyday conversation, one might expect to see a relationship between gap duration and utterance length in corpora of conversations. Thus, the experimental results suggest a new hypothesis to be tested in corpus analyses.

No corpus analyses appear to be available that specifically compared gaps before one-word and multi-word answers to polar questions. Roberts et al. (2015) reported results of statistical analyses of the gaps (floor transfer offset, FTO, in their terminology) in the Switchboard corpus (Godfrey et al., 1992), which is a large corpus of telephone conversations recorded in the United States. The FTO depended, among many other variables, on the syntactic complexity and duration of the upcoming turn. However, as shown in the Supplementary Materials of the paper, the relationship between FTO and turn duration was not linear: Overall longer turns were preceded by longer FTO, but the shortest turns (with durations below 500 ms) had the longest FTO. This is not consistent with the current findings. One can think of many reasons for this discrepancy. Most likely, the requirement to compare like with like is not met. Apart from involving different languages, the present study concerned a specific type of adjacency pairs, and all short answers were either “ja” or “nee,” whereas in the corpus analysis many different types of utterances were combined. Roberts and colleagues report that about three quarters of the short utterances were backchannels or agreements (“really?”, “hm”), whereas three quarters of the longer utterances were statements, opinions, and questions. These were not the utterance types studied here. To evaluate the conclusion from our study that long answers to polar questions are preceded by longer gaps than short answers, corpus analyses targeting these specific utterances would be needed.

Although the data reported by Roberts and colleagues cannot be used to validate our findings, we wish to draw the reader’s attention to this study for other reasons. First, it vividly illustrates the large number of variables that affect the timing of utterances in conversation and the complex relationships between them. Some variables concern the words and syntactic structures in the turns preceding and following a gap, others concern the functional roles of the turns in the sequence (e.g., whether they are initiating or responding actions), and yet others concern speaker variables, such as the interlocutors’ gender. In many cases (for instance for gender), the mechanisms through which these variables affect the timing of the utterances remain to be determined. Second, the study demonstrates that the effect of any variable that can be identified in a corpus (for instance the length of the utterances following gaps) on gap durations is likely to be very small. This is hardly surprising given the multitude of noisy and often interacting cognitive processes that are involved in listening to utterances and generating appropriate responses. To illustrate, long utterances may on average take more time to plan than short ones, and should therefore be preceded by longer gaps. However, on many occasions, speakers may be fast to initiate long utterances because of priming by the preceding context or because they only plan the first couple of words before speech onset. Conversely, speakers may often be slow to initiate short utterances because they need time to decide how to respond or are hesitant to express their views. Recordings of conversations provide very little information about these processes. Consequently, attempts to validate experimental results by comparing them broadly to the results of corpus analyses will often lead to disappointment. Combinations of experimental work and corpus analyses will be most fruitful when large, well annotated corpora are available that include sufficient numbers of the utterance types at issue in the experimental work.

Many lines of research using this approach suggest themselves. In our view, future work should not so much focus on understanding gaps between turns but rather directly address the underlying speech comprehension and planning processes. Gap durations must depend on the time the upcoming speaker requires to understand the current turn, especially the gist and speech act, the time they need to prepare a response, and the decision processes determining when to begin to plan an utterance and when to launch it (e.g., Levinson and Torreira, 2015). Each of these broadly defined components – utterance comprehension, speech planning, and the decision processes governing their coordination – should be further studied, and in each domain there are numerous specific issues to be addressed. To give a few examples, following on from the present research one could further explore the effects of presenting response-relevant information at different times in a question. The present study and the studies by Barthel et al. (2016) and Bögels et al. (2015b) showed that speakers promptly used the relevant information, but the study by Sjerps and Meyer (2015) suggested that they postponed their response planning, perhaps in order to minimize the interference between listening and speech planning. It would be useful to examine systematically when speakers initiate utterance planning as early as possible (as suggested by Levinson and Torreira, 2015), and when and why they postpone utterance planning. This would not only contribute to our understanding of conversation but also of the individual processes of speech planning and listening.

To give a second example, following on from our finding that the speech onset latencies were longer for long than for shorter utterances one could explore in more detail how far participants choose to plan their utterances before beginning to speak. There is already a rich literature on utterance planning [e.g., Konopka and Meyer (2014) for review and further references], but much of this research concerns utterances elicited in monolog settings by pictorial or written stimuli rather responses to spoken utterances. This is an important difference because in the latter situation speech planning is likely to overlap with the processing of the spoken input, and it is unknown how speakers adapt to the arising dual-task demands.

Further research should also concern social influences on listening and speech planning. There is an important line of research viewing conversation as a form of joint action (Clark, 1996; Pickering and Garrod, 2004; Garrod and Pickering, 2009; Gambi and Pickering, 2011) and stressing the importance of the interlocutors’ awareness of their joint goals and of the simulation of the partner’s behavior for smooth turn-taking (see also Brennan et al., 2010). Work related to this framework has yielded evidence for subtle social effects on speech production. For instance, Gambi et al. (2015b) showed that participants’ word production latencies were affected by their belief that another person, whom they could not see, was or was not carrying out the same task (see also Gambi et al., 2015a; Kuhlen and Abdel Rahman, 2017; Kuhlen et al., 2017). The implications of these findings for conversational turn taking need to be determined. Versions of the current paradigm could be used to do so, for instance, by assessing systematically whether social variables, such as the (assumed) presence of an interlocutor or the participants’ relationship to the interlocutor, affect speech comprehension and planning. Important goals of future work would be to determine which components of the linguistic processing system are, and which are not amenable to social influences, and through which mechanisms social influences arise.

To conclude, we advocate a two-pronged approach to research on conversation, consisting of analyses of large corpora of conversational speech paired with experimental work. To address specific research questions, the utterances extracted from the corpus and those elicited in the experiment should match as much as possible. Research along these lines should lead to a better understanding of the processes involved in listening, speaking, and their coordination and ultimately of the fine art of conversation.

## Ethics Statement

This study was carried out with written informed consent from all participants in accordance with the Declaration of Helsinki. The study was approved by the Ethics Committee of the Social Sciences Faculty of Radboud University, Nijmegen.

## Author Contributions

AM and CD designed the study. PA and CD carried out the statistical analyses. CD took the lead in writing the Methods and Results sections of the study. All the authors worked on the theoretical framing of the study.

## Conflict of Interest Statement

The authors declare that the research was conducted in the absence of any commercial or financial relationships that could be construed as a potential conflict of interest.
