# Supplementary material for: Working Together: Contributions of Corpus Analyses and Experimental Psycholinguistics to Understanding Conversation
Source: Front Psychol. 2018 Apr 12;9:525. doi: 10.3389/fpsyg.2018.00525 (PMC5906713; doi:10.3389/fpsyg.2018.00525)
Supplement: Supplementary file 1 [file Data_Sheet_1.pdf]

Appendix A: Names of objects used in Experiments 1 and 2

set 1: bijl (axe), bloem (flower), boog (bow), bril (glasses)

set 2: klok (clock), knoop (button), koets (coach), kok (chef)

set 3: plank (board), pijp (pipe), pet (cap), pan (sauce pan)

set 4: taart (cake), tak (branch), ton (barrel), trui (jumper)

Appendix B: Output model Experiment 1

Output model Experiment 1 (Linear mixed model fit by maximum likelihood)

Formula
RT ~ 1 + DisplayType * ResponseType + (1 + ResponseType + DisplayType | Participant) + (1 + ResponseType + DisplayType | Question)

Control (lmerControl(optimizer = "bobyqa"))

AIC	BIC	logLik	deviance	df.resid
31715	31814	-15840	31681	2431

Scaled Residuals

Min	1Q	Median	3Q	Max
-4.19	-0.56	-0.08	0.44	5.92

Random Effects

Groups	Name	Variance	Std.Dev	Corr	
Question	Intercept	1573	39.7		
	ResponseType[negative]	175	13.2	-.02	
	DisplayType [monochrome]	141	11.9	.48	-.28
Participant	Intercept	38316	195.7		
	ResponseType[negative]	136	11.7	.25	
	DisplayType [monochrome]	3631	60.3	.59	-.33
Residual		21819	147.7		

Fixed Effects

Effect	Estimate	Std. Error	t-value
Intercept	298	44.2	6.75
DisplayType[monochrome]	-82	13.0	-5.85
ResponseType[negative]	11	4.6	2.38
DisplayType[monochrome]:ResponseType [negative]	-28	5.3	-5.26

Note. Number of observations = 2448

Appendix C: Output model Experiment 2

Output model Experiment 2 (Linear mixed model fit by maximum likelihood)

Formula

RT ~ 1 + ResponseLength * DisplayType * ResponseType
+ (1 + ResponseType + DisplayType | Participant) + (1 + ResponseType + DisplayType | Question)

Control (lmerControl(optimizer = "bobyqa"))

AIC	BIC	logLik	deviance	df.resid
38362	38487	-19160	38320	2823

Scaled Residuals

Min	1Q	Median	3Q	Max
-2.79	-0.66	-0.15	0.51	4.52

Random Effects

Groups	Name	Variance	Std.Dev	Corr	
Question	Intercept	21330	146.0		
	ResponseType[negative]	1160	34.1	.29	
	DisplayType[monochrome]	1362	36.9	.61	.13
Participant	Intercept	1.839	42.9		
	ResponseType[negative]	0.4	0.7	-.29	
	DisplayType[monochrome]	2	1.3	.88	-.71
Residual		37940	194.8		

Fixed Effects

Effect	Estimate	Std. Error	t-value
Intercept	538	25.8	20.88
ResponseLength[Long]	139	23.4	5.93
DisplayType[monochrome]	-56	7.0	-7.97
ResponseType[negative]	37	6.7	5.56
ResponseLength[Long]:DisplayType [monochrome]	25	7.0	3.59
ResponseLength[Long]:ResponseType [negative]	11	6.7	1.70
DisplayType[monochrome]:ResponseType [negative]	-18	3.9	-4.58
ResponseLength[Long]:DisplayType [monochrome]:ResponseType[negative]	-2	3.9	-0.60

Note. Number of observations = 2844

Appendix D: Comparison of Experiments 1 and 2

Output model Experiment 1 vs short responses Experiment 2 (Linear mixed model fit by maximum likelihood)

Formula
RT ~ 1 + exp * DisplayType * ResponseType
+ (1 + ResponseType + DisplayType | Participant:exp) + (1 + ResponseType + DisplayType | Question:exp)

Control (lmerControl(optimizer = "bobyqa"))

AIC	BIC	logLik	deviance	df.resid
50320	50452	-25139	50278	3830

Scaled Residuals

Min	1Q	Median	3Q	Max
-3.97	-0.59	-0.10	0.45	5.61

Random Effects

Groups	Name	Variance	Std.Dev	Corr	
Question	Intercept	1599	40.0		
	ResponseType[negative]	134	11.6	-.14	
	DisplayType [monochrome]	99	9.9	.45	-.25
Participant	Intercept	28068	167.5		
	ResponseType[negative]	209	14.5	.07	
	DisplayType [monochrome]	3100	55.7	.62	-.29
Residual		24527	156.6		

Fixed Effects

Effect	Estimate	Std. Error	t-value
Intercept	348	27.2	12.79
exp[1]	-50	27.2	-1.84
DisplayType[monochrome]	-81	9.3	-8.70
ResponseType[negative]	19	4.0	4.67
exp[1]:DisplayType[monochrome]	0	9.4	-0.03
exp[1]:ResponseType[negative]	-8	4.0	-1.90
DisplayType[monochrome]:ResponseType [negative]	-22	3.5	-6.16
exp[1]:DisplayType[monochrome]: ResponseType[negative]	-6	3.5	-1.76

Note. Number of observations = 3851

The null effect for the interactions between Experiments and DisplayType and between Experiments and Response Type suggests that the manipulation effects were comparable across experiments. This conclusion is supported by an additional Bayesian analysis with very weakly informative priors (N(0,500) for all fixed effects, i.e. assuming that 95% of effects will lie in the range -1000ms to +1000ms) using Stan and the R package brms (Bürkner, 2017). This analysis, with Bayes Factors all above 20 for the lack of an interaction, is more directly suggestive of there being no interaction between Experiment and any manipulation. For the potential main effect of Experiment (est=-50ms, t=-1.84 in the combined frequentist model; Bayesian credible interval [-108.70, 6.73]), the statistical interpretation is clear: Participants in Experiment 1 may have been faster overall than in Experiment 2, but the evidence for this statement is mixed..

Bayesian hypothesis testing of null-hypothesis for interaction terms with Experiment

Interaction Term	Est.	Err.	CI l-95%	CI u-95%	Bayes Factor for Point Null Hypothesis
exp1:Conditionmonochrome	-0.21	9.80	-19.68	18.74	54.45
exp1:TargetAnswernegative	-7.59	4.08	-15.67	0.48	23.61
exp1:Conditionmonochrome:TargetAnswernegative	-6.34	3.58	-13.25	0.75	31.47

Reference:

Bürkner, Paul-Christian (2017). brms: An R Package for Bayesian Multilevel Models Using Stan. Journal of Statistical Software, 80(1), 1-28. doi:10.18637/jss.v080.i01
